# Supplementary material for: Pharmacological Interaction of Quercetin Derivatives of Tilia americana and Clinical Drugs in Experimental Fibromyalgia
Source: Metabolites. 2022 Sep 28;12(10):916. doi: 10.3390/metabo12100916 (PMC9607183; doi:10.3390/metabo12100916)

Supplementary Material

Since D_2 dopamine and μ opioid receptors and/or serotonin transporter are not molecular targets for TRA or PRA, respectively, it was not included the docking analysis of these interactions in the manuscript. However, these results are considered in this section of supplementary material to demonstrate their low probability of interaction. To support the possible direct interaction of quercetin on the receptors of TRA and PRA such as μ-opioid, serotonin transporter and D_2 dopamine receptors. The crystal structure of the compounds TRA and PRA were obtained from the PubChem database, these structures were protonated using the Avogadro software for a pH of 7.4 and subsequently the minimum energy spatial configuration was determined using the the Merck Molecular Force Field (MMFF94). The protein structure of mu opioid (4DRL), serotonin transporter (6VRH) or D_2 (6CM4) dopamin receptors was obtained from the Protein Data Bank (PDB, https: //www.rcsb.org/), for each case a resolution of ≤3.3 Å was selected. Then, docking was performed by the CB-Dock tool, results from CB-Dock tool software were contrasted with UCSF Chimera 1.16 for protein preparation and Autodock vina 1.1.2.

The PRA-μ-opioid receptor and PRA-serotonin transporter docking showed non-steric interactions like those observed with the TRA-μ-opioid complex or TRA-serotonin transporter complex in the manuscript. Based on the binding energy results, it can be predicted that these three interactions are unlikely to occur due to the high binding energies. As observed in panel A, TRA shows a binding energy in the range of −6.4 to −5.8 kcal/mol for the dopamine D_2 receptor, similar to that obtained for PRA for the μ opioid receptor (−6.4 to 5.8 kcal/mol) in panel B. Whereas, an interval of −6.7 to −5.0 kcal/mol was calculated for PRA for the serotonin transporter in panel C. It is observed that tramadol and the D_2 dopamine receptor complex shared 2 steric interactions compared to tramadol-μ-opioid complex (ASP114 and TYR416).

A

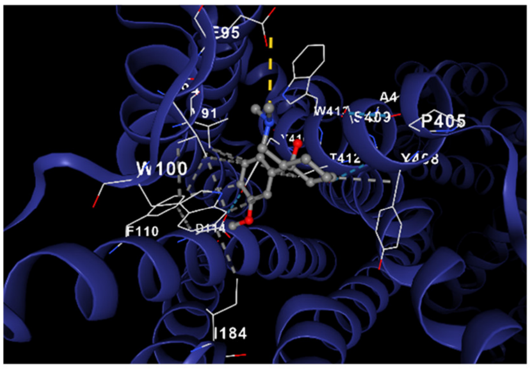

B

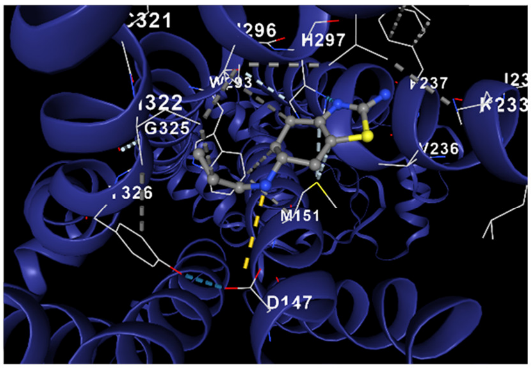

C

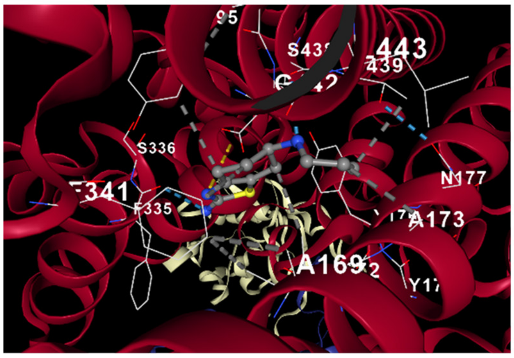

Supplement: Supplementary file 1 [file metabolites-12-00916-s001.zip › metabolites-1875471-supplementary.pdf]
